# Mechanical Properties and Dimensional Stability of Poplar Wood Modified by Pre-Compression and Post-Vacuum-Thermo Treatments

**DOI:** 10.3390/polym14081571

**Published:** 2022-04-12

**Authors:** Zaixin He, Yanran Qi, Gang Zhang, Yueying Zhao, Yong Dai, Baoxuan Liu, Chenglong Lian, Xiaoying Dong, Yongfeng Li

**Affiliations:** 1 Key Laboratory of State Forestry Administration for Silviculture of the Lower Yellow River, College of Forestry, Shandong Agricultural University, Tai’an 271018, China; hzaixin@126.com (Z.H.); qyran1994@163.com (Y.Q.); zhanggang0322@163.com (G.Z.); 2 Postdoctoral Innovation Practice Base, Shandong Xiaguang Group Co., Ltd., Jining 277600, China; xgsyyf@126.com; 3 Jiangsu Longyuan Decoration Materials Co., Ltd., Suqian 223900, China; yongdaily@126.com; 4 Shandong Laucork Development Co., Ltd., Jining 272100, China; liubaoxuan123@126.com

**Keywords:** compression, vacuum-thermo treatment, mechanical properties, dimensional stability, poplar wood

## Abstract

Fast-growing poplar wood has the bottleneck problems of inferior mechanical strength and poor dimensional stability. In this study, the wood was modified by combined treatments of pre-compression and post-vacuum-thermo modification to improve its mechanical strength and dimensional stability, simultaneously; in addition, the variation law of mechanical properties of the wood with compression ratio as well as the improvement effect of dimensional stability of the treated wood were mainly studied. The results show that the optimal temperature and time of the vacuum-thermo modification were 190 °C and 10 h, respectively. Under these conditions, the structure of pre-compressed and post-vacuum-thermally modified wood (CT wood) is gradually densified with the increase in the compression ratio, which results in the continuous enhancement of mechanical properties. Meanwhile, the anti-swelling efficiency (ASE) of the CT wood after water absorption is correspondingly better than that of the compressed wood before thermal modification, indicating that the dimensional stability of compressed wood was improved by the thermal modification. When the compression ratio was 70%, the modulus of rupture (MOR) and impact toughness of CT wood was 176 MPa and 63 KJ/m^2^, which was 125% and 59% higher than that of untreated wood, respectively. The ASE was also 26% higher than that of the wood with sole compression. Therefore, this method improves the mechanical strength and dimensional stability of wood simultaneously, and it provides a scientific basis for optimization of the reinforcing modification process of fast-growing wood.

## 1. Introduction

Forest resources play a fundamental role in regulating ecology, improving climate, and providing abundant and renewable woody resources as a substitute to non-renewable resources, such as steel and plastic, to reduce the environmental pollution caused by their processing and utilization [1]. Therefore, fully utilizing wood resources aids in the green and sustainable development of the economy and society. However, trees grow slowly, resulting in a long period before initially providing wood materials, especially for high-quality wood resources, which makes it difficult to meet the urgent demand for wood resources supplied for rapid economic and social development. Although fast-growing trees have alleviated the tight supply of wood resources, they have the drawbacks of lower strength and unstable dimension of moisture absorption and water absorption, particularly for wood materials such as poplar wood (*Populus* L.), which restricts the wider applications of such resources as substitutes for non-renewable resources [2,3,4].

Various modification methods have been explored and applied to fast-growing wood for simultaneous improvement in mechanical strength and dimensional stability such as resin impregnation [5,6,7], densification combined with pretreatment of partial lignin removal [8], chemical modification with low molecular agents [9], and a combination of densification and thermal modification [10,11,12,13,14,15]. However, until now, the combination of densification and thermal modification has only been in line with the concept of sustainable development. Compression treatment is used to densify wood, mainly through pressure combined with/without hydrothermal treatment to improve its mechanical strength [15,16]. Thermal modification mainly degrades and converts partially hydrophilic hemicelluloses into hydrophobic substances to improve their dimensional stability through high temperature with/without inert gas protection [17,18,19]. The extent of these changes in wood during the heat treatment depends on the thermal modification method, the wood species and its typical properties, the initial moisture content of the wood, the surrounding atmosphere, treatment time, and temperature [20]. Combining the two methods could simultaneously improve the mechanical strength and dimensional stability of wood [10,11]. The whole process does not involve chemical substances that pollute the environment or require high energy consumption during the manufacturing process. The wood modification mainly relies on the hydrothermal effect with lower energy consumption, which is environmentally friendly, green, low carbon and, thus, has been widely explored. For example, the mechanical strength and dimensional stability of wood could be improved simultaneously by the following treatments: combined segmented heating and hot-pressing treatments [12], combined hot steam and compression treatments [16], combined pre-compression and post-hot oil modification [21], combined pre-compression and post-nitrogen-protected thermal modification [22]. 

However, due to the variety of the heat treatment methods, they often have different degrees of negative effects on the mechanical strength of the treated wood. Okon’s [23] research found that under the condition of treatment at 210 °C for 4 h, the mechanical properties of silicone oil thermally modified Masson pine wood were two times lower than those of untreated samples. Pelit [24] studied the effect of densified and heat post-treated on the mechanical properties of wood. The results showed that the MOR values of Uludağ fir, linden, and black poplar wood were reduced by 48%, 56%, and 39%, respectively, when they were compressed at a 100 °C and 50% compression ratio and then heat treated at 212 °C. Bal [25] studied the thermal modification of black pine wood at 180, 200, and 220 °C for 150 min in vacuum, nitrogen, and air atmospheres. The results showed that with the increase in the modification temperature, the loss of mechanical properties was greater. At 220 °C, the MOR of thermally modified wood in air, vacuum, and nitrogen atmospheres decreased by 23.8%, 12.9% and 19.8%, respectively. Therefore, it is of great practical significance to promote wide application of the combined densification and thermal modification treatment by selecting an appropriate thermal modification method to reduce the decline in the mechanical strength.

**Figure 1:**
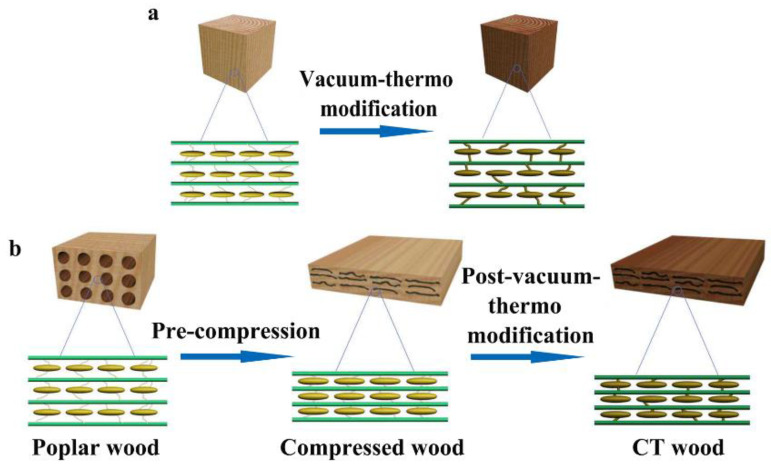
Schematic diagram of wood modification with combined pre-compression and post-vacuum-thermo treatments: (**a**) vacuum-thermo modification; (**b**) combined pre-compression and post-vacuum-thermo treatment.

High-temperature thermal modification partially degrades hemicellulose into volatile substances with small molecules, such as organic acids, aldehydes, and furans, which reduces the mechanical properties. Among them, acidic small molecules could accelerate the degradation of polysaccharide macromolecules and, thus, adversely reduce the mechanical strength of wood [26,27,28]. Therefore, during thermal modification, timely removal of such small molecular substances by proper methods, such as vacuum treatment, is helpful for protecting the mechanical properties of the compressed wood. Therefore, combing the two ways by pre-compression and post-vacuum-thermo modification could theoretically obtain high-quality poplar wood (CT wood) with improved mechanical properties and desired dimensional stability [10]. However, such treatment has rarely been reported. Therefore, this study employed this method to modify wood with a focus on optimizing the process of vacuum thermal modification and the variation laws of wood mechanical properties and dimensional stability with the compression rate under the optimized thermo modification in order to provide a scientific basis for the wider application of such treatment (Figure 1).

## 2. Materials and Methods

The poplar wood board employed in this study was bought from the wood market in Tai’an city, Shandong Province. The contents of hemicellulose and lignin in poplar wood are about 21% and 24%, respectively [29,30]. Temperature and time were explored as the main indexes for the optimization of vacuum thermal modification conditions. Four temperatures (i.e., 170, 180, 190, and 200 °C) and six thermal modification times (5, 10, 15, 24, 36, and 48 h) were explored. The experiment was designed by the all-factor method, and three parallel groups of data were tested to obtain the average value for evaluating each physical property. Firstly, the samples were dried with a drying oven (DZF-6050, Shanghai Huitai Analytical Instrument Co., Ltd., Shanghai, China) at 103 °C for 48 h; then, vacuum treatment was conducted in the oven with a vacuum pump until the absolute pressure reached 0.01 MPa; after, the temperature was raised to the explored values and kept for the whole thermal modification treatment. Finally, the samples were naturally cooled to room temperature under the vacuum condition.

The treatment of pre-compression and post-vacuum-thermo modification was conducted as follows. Poplar blocks of 100 × 100 × 20 (L × T × R mm^3^) were employed and placed in a water bath (HH-2, Jintan Baita Xinbao Instrument Factory, Changzhou, China) at 90 °C for 2 h to soften the wood components. Then, the softened samples were further compressed by a hot-pressing machine (YLJ-HP88V, Hefei Kejing Material Technology Co., Ltd., Hefei, China) for different compression ratios of 30%, 50%, and 70%. The total pressing temperature was first set to 60 °C and maintained for 1 h, and then it was raised to 100 °C for 4 h. Next, the wood blocks were moved to a drying oven and dried at 103 °C for 48 h. Finally, the wood samples were vacuumed at 0.01 MPa and hot treated under the optimized thermal modification conditions (i.e., temperature and time). Before further evaluation of the properties, all samples were naturally cooled to room temperature under the vacuum condition and placed under a ventilation condition for more than 1 week.

Density was calculated from the sample’s volume and weight. The dimensional stability was evaluated mainly based on the anti-swelling efficiency (ASE) of wood after moisture or water absorption [26]. The treated wood samples were put into a sealed tank under different moisture levels or into water solution, and then taken out after moisture/water treatment for 2, 5, 10, 24, 48, 72, 96, and 120 h, respectively. Each sample volume change was finally calculated in terms of the sample volume before and after moisture/water treatment [26]. Hardness, modulus of rupture (MOR), and impact toughness were measured using a universal mechanical testing machine (CMT4104, Xinsansi Material Testing Co., Ltd., Shenzhen, China), respectively, according to the GB/T 1929-2009 and GB/T 1936.1-2009. The thermogravimetric (TG) behavior was characterized by a synchronous thermal analyzer (TGA Q500, TA company, Boston, MA, USA). The microstructure, component, and surface elements of the samples were characterized by SEM (JEM-6610LV, JEOL, Akishima, Japan), XRD (D/MAX 2200, Rigaku, Tokyo, Japan), FTIR (Nicolet Magna 560, ThermoFisher Scientific, Waltham, MA, USA), and XPS (STA 449F3, ThermoFisher Scientific, American).

## 3. Results and Discussion

### 3.1. Optimization of Vacuum Thermal Modification Process

#### 3.1.1. Density

**Figure 2:**
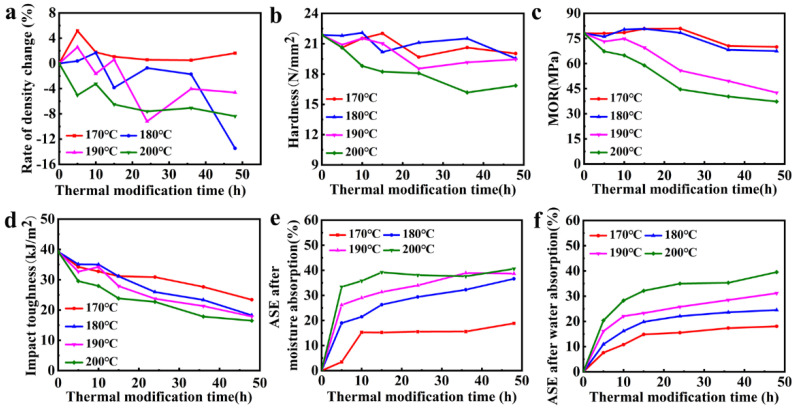
The (**a**) density change rate; (**b**) hardness; (**c**) modulus of rupture; (**d**) impact toughness of thermally modified wood at different temperatures; the ASE of wood after (**e**) moisture absorption at 98% humidity and (**f**) after water absorption with only thermal treatment.

Figure 2a shows that the wood density, overall, decreased with the thermal modification time and temperature. The density change was mainly due to the mass change caused by component degradation, while the volume was basically unchanged in this temperature and time range. This is the pyrolysis of hemicellulose in the amorphous region and the rapid pyrolysis of lignin, which formed charcoal and volatile intermediates and decreased the wood density. At temperature of 170 °C, the change in wood density was not obvious during the entire treatment period, which indicates that such temperature and time had no significant effect on wood density. Within the range of 180 °C and 200 °C, the wood density decreased obviously with the thermal modification time; especially at 200 °C, the density declined obviously during the entire experimental period, indicating that the wood components were degraded continuously at this temperature, which would have an obviously negative impact on the mechanical properties of wood. Within the temperature range of 180 °C and 190 °C, the wood density remained stable during the treatment stage in the first 10 h, and began to decrease significantly after 15 h of treatment, indicating that the wood components were not significantly degraded within treatment time of 10 h, which was conducive to the maintenance of the wood’s mechanical strength. These results match research by Hajihassani [12], Bal [25], and Čabalová [27], showing the same variation law.

#### 3.1.2. Mechanical Properties

Figure 2b–d show that the hardness, modulus of rupture, and impact toughness of the wood decreased with the thermal modification temperature and time. At the treatment temperature of 200 °C, the three mechanical strengths continued to decrease with the thermal modification time, indicating that this temperature could adversely affect the mechanical properties. Under thermal modification, pyrolysis of hemicellulose in the amorphous region and rapid pyrolysis of lignin occurred, resulting in reduced mechanical properties. At the same time, with the prolongation of the thermal modification time, the degradation of hemicellulose and lignin increased; thus, there was a continuous downward trend. When the treatment temperature was in the range of 170 °C and 190 °C, the mechanical strength fluctuated in varying degrees during the first treatment time of 10 h, but there was no obvious decrease overall. This may be due to the fact that the initial wood had a certain amount of moisture or other substances that absorbed the heat during thermal modification and delayed the degradation time. As the moisture or other substances evaporated or dissipated, the wood gradually reached the thermal modification and then the mechanical properties began to decline in varying degrees after 10 h. This was basically consistent with the variation law of wood density, indicating that change in density affected the mechanical strength and, subsequently, both were positively correlated [28,31]. The decrease in MOR may mainly be due to the degradation of hemicellulose, which weakens the fixation of cellulose chain [26,32,33]. Figure 2e,f show that both the ASE after moisture absorption and water absorption increased with the vacuum temperature; in other words, the dimensional stability was positively correlated with the thermal modification temperature. During the early treatment time of 10–15 h, it increased significantly with the treatment time and then plateaued, indicating that the effect of thermal modification time on ASE was mainly restricted within the first 15 h [28]. This was probably due to the degradation of hydrophilic chemical components, such as hemicellulose, after thermal modification; the reduction in water absorption capacity; the increase in ASE. At approximately 15 h, with the completion of the degradation of a large amount of hemicellulose, the change of ASE also became flat.

#### 3.1.3. Optimum Thermal Modification Process

Totally, the temperature of 200 °C had the greatest improvement in dimensional stability, however, it had a largely negative effect on the mechanical strength of wood. While in the temperature range of 170 °C and 190 °C, the best dimensional stability was attained at 190 °C. Under this temperature and during the thermal modification time of the early 10 h, the mechanical strength of wood did not decrease significantly but only fluctuated slightly. After a period of time, the overall strength of the wood began to decline significantly. Similarly, the wood’s dimensions become stable after the 10 h. Therefore, combining the mechanical strength and dimensional stability, 190 °C and 10 h were employed as the optimal conditions for vacuum thermal modification. This temperature was the same as the optimal thermal modification temperature reported in the literature [11,20,25].

#### 3.1.4. SEM

**Figure 3:**
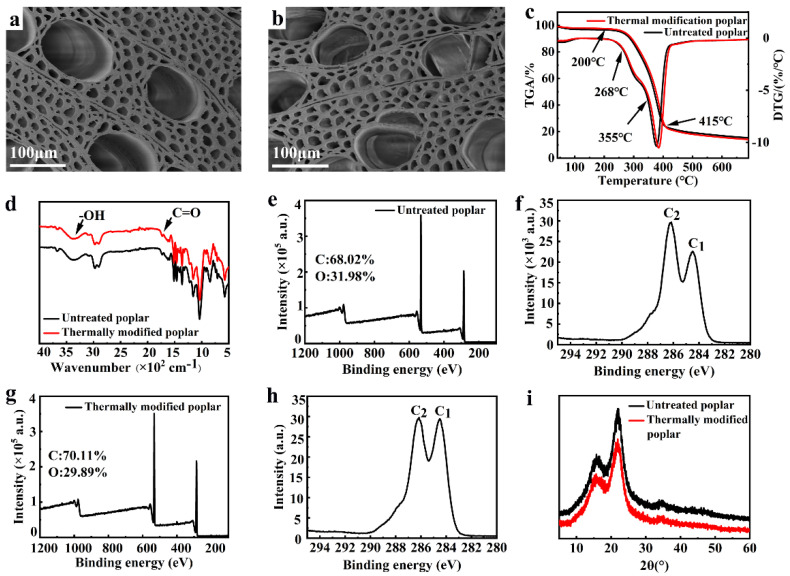
SEM morphologies of the (**a**) untreated wood and the (**b**) thermally modified wood; (**c**) TG curves of the untreated wood and the thermally modified wood; (**d**) FTIR spectra; XPS spectra of (**e**) untreated woods (**f**) the carbon element peak of untreated wood, (**g**) thermally modified wood, and (**h**) carbon element peak of the thermally modified wood; (**i**) X-ray diffraction pattern of untreated wood and the thermally modified wood with the temperature of thermal treatment of 190 °C.

Figure 3a,b show that the modified cell wall did not change significantly and still maintained a stable cell arrangement structure, which was almost the same as that of the untreated wood. Therefore, the thermal modification treatment at 190 °C for 10 h could not destroy the integrity of the cell wall structure, which further proved that the physical and mechanical properties of wood did not decrease significantly after the thermal modification [34].

#### 3.1.5. Thermogravimetry

Figure 3c shows that the thermal degradation mainly occurred in the temperature range of 200 °C and 415 °C, hemicellulose and cellulose began to degrade at 200 °C and 250 °C, respectively, and the maximum loss rate appeared at 268 °C and 355 °C. The thermal degradation temperature of lignin started from 180 °C [35,36,37]. This was the pyrolysis of hemicellulose in the amorphous region and the rapid pyrolysis of cellulose and lignin, which formed charcoal and volatile intermediates (CO_2_, CO, CH_4_, CH_3_OH, CH_3_OOH, hydrocarbons, hydroxyl compounds, carbonyl compounds, etc.) [38,39]. Comparing the TG curves of untreated wood and thermally modified wood, it can be observed that the onset temperature of the thermally modified wood was similar to the peak temperature of weight loss, which was slightly higher than that of untreated wood, indicating partial degradation of hemicellulose or structural rearrangements caused by volatilization of some substances in wood during the thermal modification process.

#### 3.1.6. FTIR Spectra

Figure 3d depicts that the absorption peak of the thermally modified wood near 3363 cm^−1^ corresponded to the stretching vibration of the −OH group, and the absorption peak intensity of the thermally modified wood decreased slightly compared to the untreated wood, indicating that the number of hydroxyl groups in the wood decreased to a certain extent after thermal modification. The hemicellulose has abundant and active hydroxyl groups that can condensate at higher temperatures to form −O− bonds, removing water molecules and thereby eliminating part of the free hydroxyl groups [40]. Additionally, hydroxyl and carbonyl groups of various components in wood cell walls could strongly interact with each other by hydrogen bonds or van der Waals force at high temperatures, which further reduced the number of free hydroxyls groups [41].

The absorption peak near 1731 cm^−1^ corresponded to the stretching vibration of the C=O group, which was mainly originated from the acetyl and carboxyl groups of hemicelluloses. The absorption peak of wood slightly decreased after the thermal modification, which was mainly due to the pyrolysis of hemicellulose and the change in chemical composition in the wood during the process [42]. The acetyl groups of hemicellulose were cleaved at high temperatures and underwent a deacetylation reaction with free hydroxyl groups to form acetic acids. With the increase in temperature, the pyrolysis of hemicellulose was further promoted, resulting in a decrease in the number of hydroxyl groups; some polysaccharides in hemicellulose were cleaved into furfural or certain carbohydrates with short-chain structures, which further polymerized into some water-insoluble polymers under the high temperatures [34,40]. Nicolas believed that the thermal modification process was accompanied by the cleavage of lignin β-aryl-ether bonds, followed by condensation reactions and cross-linking reactions between lignin biopolymers that formed a novel cross-linked network structure [43].

#### 3.1.7. X-ray Photoelectron Spectroscopy

The elemental composition of the wood before and after thermal modification were determined by X-ray photoelectron spectroscopy (XPS), and the results are shown in Figure 3e–h. There were no novel peaks presented in the thermally modified wood, indicating that new elements were absent in the process. The content of carbon element increased after thermal modification, which was due to the decrease in the oxygen element content caused by the decrease in the number of hydroxyl groups [40]. The increase in C_1_ content mainly resulted from the reaggregation of lignin components, which is consistent with the results in Figure 3d.

#### 3.1.8. X-ray Diffraction Pattern

Figure 3i shows that the diffraction peak of the I_002_ crystal plane of the thermally modified wood was still located near 22°, indicating that the cellulose crystalline was stable without crystal form change during the treatment. However, the crystallinity decreased from 49% to 44%. This was mainly due to the acetyl groups on hemicellulose fall off and acetic acids formed, which resulted in the partial acidolysis of the cellulose molecular chain at high temperatures, and further destroyed the cellulose aggregation to reduce the polymerization degree of cellulose and, accordingly, led to the crystallinity reduction [43]. A previous study reported that the crystallinity of larch wood decreased at 230 °C under nitrogen atmosphere as protective gas [44]. This result explains, to a certain extent, the decline in the physical and mechanical properties of the thermally modified wood.

### 3.2. Combining Pre-Compression and Post-Vacuum-Thermo Treatments

#### 3.2.1. SEM

**Figure 4:**
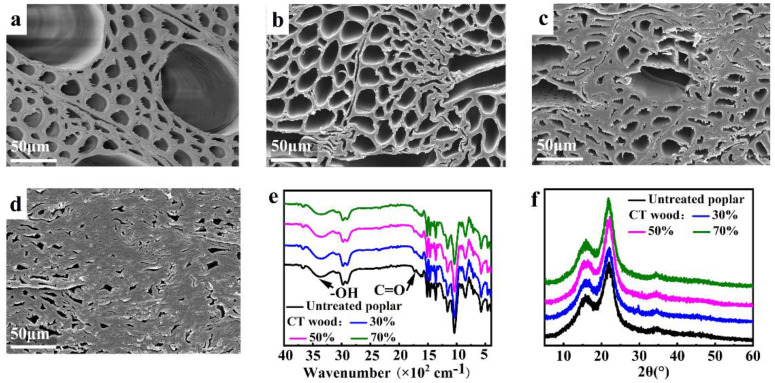
SEM of (**a**) untreated wood; CT wood with a (**b**) 30%, (**c**) 50%, and (**d**) 70% compression ratio; (**e**) FTIR spectrum of untreated wood and CT wood with different compression ratios; (**f**) X-ray diffraction patterns of untreated wood and CT wood with different compression ratios.

Based on the above optimized thermal modification, the poplar wood was modified by the combined treatments of pre-compression and post-vacuum-thermo modification, and the variation law of mechanical properties and dimensional stability of the wood with the compression ratio were focused on and explored. The internal microstructures of CT wood were observed by SEM to analyze the changes in the physical and mechanical properties. Figure 4a–d show that the diameter of the cell lumen gradually decreased with the increase in the compression ratio, that is, the porosity gradually decreased, and the wood cell wall accordingly became denser. A previous study [45] described that the cell structure, in a cross-section, was distorted but was not significantly damaged after compression, which intuitively explains the density increase of the wood caused by compression and, therefore, improved the hardness, bending strength, and impact toughness. Additionally, the densified structure also demonstrated that the improvement in the mechanical properties caused by compression treatment was greater than the negative effect of the thermal modification on the mechanical properties [45,46].

#### 3.2.2. FTIR Spectra and X-ray Diffraction Pattern

Figure 4e shows that the absorption peak intensity of CT wood near 3363 cm^−1^ (−OH group) was reduced, indicating a reduction in the −OH group that was mainly caused by the degradation of hemicellulose during the thermal modification process. The absorption peak decreased at 1731 cm^−1^ (C=O group), further confirming the degradation of hemicellulose. Figure 4f describes that the diffraction peak of the I_002_ crystal plane was still located near 22° after the combined treatments, indicating that the cellulose crystal form did not change without dissolution or chemical treatment [47]. The crystallinity of the untreated wood and the CT wood with compression ratios of 30%, 50%, and 70% was 44%, 46%, 49%, and 49%, respectively. With the increase in the compression ratio, the crystallinity also increased. During the compression process, the distance between the cellulose fibrils in the amorphous region was reduced so that the fibrils were arranged more closely; the transverse compression caused the fibrils to only move in the cross-section, which aided the fibrils orientally arranged along the direction of the crystalline region [48,49,50]. In addition, the thermal modification itself removed partial hemicellulose in the amorphous region, which promoted the improvement in the crystallinity. All these effects will comprehensively contribute to the improvement in the mechanical properties of wood.

#### 3.2.3. Density

**Figure 5:**
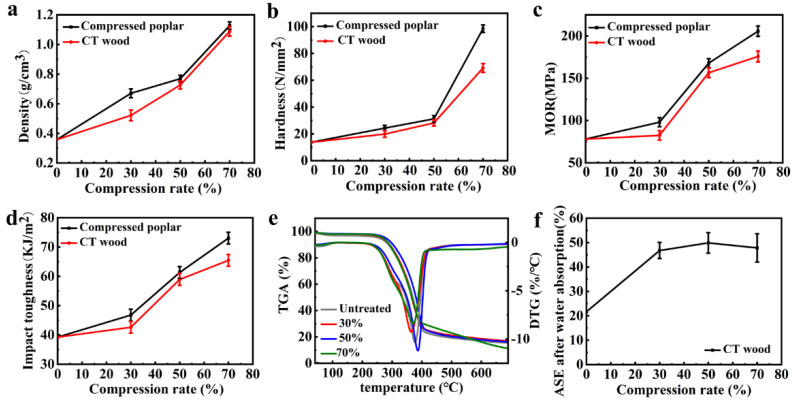
Results of (**a**) density; (**b**) hardness; (**c**) MOR; (**d**) impact toughness; (**e**) TG curves; (**f**) ASE after the water absorption of the CT wood.

Figure 5a shows that the density of the wood increased almost linearly with the compression ratio. When the ratio reached 70%, the poplar wood’s density increased by approximately two times. Although the density of the compressed wood decreased after the post-vacuum-thermo modification due to the degradation of hemicellulose, there was no significant change. When the compression ratio was 70%, the thermal modification treatment only reduced the wood’s density by 4%. This indicates that such a combined treatment could effectively reduce the density change in wood and, accordingly, promote the dimensional stability and mechanical properties of the modified wood [51,52,53].

#### 3.2.4. Mechanical Properties

Figure 5b shows that the hardness of the compressed wood without post-thermal modification increased significantly with the compression ratio, and the maximum was approximately six times that of the uncompressed wood (i.e., untreated wood) [51,53,54]. An increase in the compression ratio resulted in an increase in density, which led to the significant increase in hardness. The hardness of the CT wood was slightly lower than that of the compressed wood, but the decrease in the compressed wood, reduced by thermal modification, was not obvious when compared to the hardness increase in the wood caused by compression. When the compression ratio reached 70%, the maximum hardness was 69 N/mm^2^, which was four times that of the original wood. Similar to the above hardness change, all the MOR and impact toughness of the compressed wood increased with the compression ratio, no matter before and after the post-vacuum-thermo treatments (Figure 5c,d); when the compression ratio was 70%, the MOR and impact toughness attained 206 MPa and 73 KJ/m^2^, which was 164% and 86% higher than that of the untreated wood, respectively [55,56]. In the same way, the MOR and impact toughness of the CT wood was slightly lower than that of the compressed wood without the post-thermal modification, respectively. However, compared with the increase in the MOR and impact toughness of the compressed wood, the decrease of both values of the CT wood reduced by thermal modification was not significant. When the compression ratio reached 70%, the MOR and impact toughness still achieved 176 MPa and 63 KJ/m^2^, which was 125% and 59% higher than that of the untreated wood, respectively. With the progress in compression, the arrangement of cellulose fibers became closer and the interaction force became stronger; the thermal modification removed partial hemicellulose in the amorphous region, and the compression process induced a more oriented fiber arrangement, resulting in a relative increase in crystallinity; therefore, the mechanical properties showed a slope-increasing improvement [10,11,12,14]. Consequently, combining pre-compression and post-vacuum-thermo treatments could significantly improve the hardness, bending strength, and impact toughness of wood [24,57].

#### 3.2.5. Thermogravimetry

In addition, the maximum thermal degradation temperature of CT wood also changed, to a certain extent, with the increase in the compression ratio (Figure 5e). The maximum degradation temperature was 9 °C higher than that of untreated wood at a compression ratio of 50%. When the compression ratio was 30% and 70%, it was slightly lower than that of untreated wood, respectively, but the difference was not significant. From what is shown, we can see that the combined treatments of pre-compression and post-vacuum-thermo modification had little significant effect on the thermal stability of wood. 

#### 3.2.6. ASE

Figure 5f showed that all the ASE of the compressed wood with different compression ratios were improved after the thermal modification. Especially, the ASE increase of CT wood with compression ratio of 50% was 28% higher than that of uncompressed and the only thermally modified wood. Therefore, thermal modification improved the dimensional stability significantly, especially for the compressed wood [34].

In short, combining pre-compression and post-vacuum-thermo treatments effectively and simultaneously improved the mechanical properties and dimensional stability of wood, which was the expected outcome of the study. Generally, with increase in the compression ratio, the mechanical properties of the wood improved, while the ASE declined, and the dimensional stability accordingly decreased. In this study, with the thermal modification combined the pre-compression, bending strength, and impact toughness of the CT wood increased significantly with the compression ratio; meanwhile, it maintained a satisfied ASE, indicating a simultaneously improved dimensional stability of the wood [14,45,58].

## 4. Conclusions

Focusing on the defects of poor mechanical properties and dimensional stability of poplar wood caused by its soft substrate, this study explored the method of combining pre-compression and post-vacuum-thermo modification and analyzed the variation law of mechanical properties of CT wood with different compression ratios and the improvement in the dimensional stability. The conclusions are as follows:

1. With the increase in thermal modification temperature and time, hemicellulose and other substances gradually decomposed and changed, resulting in decreases in the mechanical properties of wood and improvement in the dimensional stability of wood. Considering both the mechanical properties and dimensional stability, 190 °C and 10 h were determined as the optimum conditions for thermal modification;
2. With the increase in the compression ratio, the cell porous structure of the poplar wood became denser, which improved the density, hardness, MOR, and impact toughness but adversely affected the dimensional stability. After further treatment with thermal modification, all of the poplar wood with different compression ratios presented improved ASEs and a reduced negative impact on the mechanical properties. The MOR and impact toughness of CT wood at a compression ratio of 70% was 176 MPa and 63 KJ/m^2^, which was 125% and 59% higher than that of untreated wood, respectively. The ASE of the CT wood was 26% higher than that of the only compressed poplar wood. Consequently, such a method could improve the mechanical properties and dimensional stability of poplar wood simultaneously and significantly, and it can be applied to floors, load-bearing walls, etc. Further research on larger-scale poplar and other wood species will provide a scientific basis for large-scale application of this method.
